# Maximal androgen blockade for advanced prostate cancer

**DOI:** 10.4103/0970-1591.60463

**Published:** 2010

**Authors:** Rajiv Paul Mukha, Santosh Kumar, N. S. Kekre

**Affiliations:** Department of Urology, Christian Medical College Vellore, Vellore - 632 004, India

**Keywords:** Carcinoma prostate, maximum androgen blockade, androgen suppression

## Abstract

Prostate cancer has now become one of the leading types of cancer in urban India. It is now the third most common cancer in Delhi. As we advance in health care with the resultant increase in longevity, we will be seeing more of advanced carcinoma prostate. Since the early 1980.s, there have been many trials on MAB. However, the question remains whether these agents actually make a difference? The role of MAB is probably limited to the prevention of the β are reaction in patients on LHRH agonists. The non steroidal antiandrogens have a marginal benefit of increased overall survival by approximately 3% to 5% at 5 ve years. There may be a role for MAB in patients with metastatic carcinoma of prostate, low volume metastases, patients with M 1 disease with absence of metastases in the skull, ribs, long bones, and soft tissues excluding lymph nodes.

## INTRODUCTION

Prostate cancer has always been considered a disease of the West. The incidence of prostate cancer has been on the rise as evidenced by various urban population based registries.[1] Prostate cancer has now become one of the leading types of cancer in urban India. It is now the third most common cancer in Delhi.[2] As we advance in health care with the resultant increase in longevity, we will be seeing more of advanced carcinoma prostate. The 1994 United States Medicare expenditures for the treatment of prostate cancer were almost 1.5 billion dollars. A large proportion of this expense was associated with the use of anti-androgen interventions. Their justification, hinges on long standing observations about the responsiveness[3] of prostate cancer to androgen suppression. In our country where health is paid for by the individual, it is important for us to have an accurate knowledge of the facts before prescribing to expensive therapies.

In advanced prostate cancer, the treatment is androgen suppression surgically or medically. Testosterone from the testis provides most, but not all the androgenic activity can be eliminated with androgen suppression (AS). The low plasma concentrations of androgens that remain after AS are of adrenal origin and may still have some stimulatory effect on any hormone-sensitive parts of the prostate cancer. Huggins and Scott[4] first examined this in 1945 by performing bilateral adrenalectomies on patients with carcinoma of prostate.

In the1980s, Labrie hypothesized that counteracting adrenal androgens would further inhibit the growth of tumor and possibly improve symptoms and survival beyond the response achieved with monotherapy. This residual effect can be suppressed by the addition of an antiandrogen like nilutamide, flutamide, cyproterone acetate or bicalatumide. Such combination of AS with an antiandrogen is referred to as maximum androgen blockade (MAB).

Since the early 1980's, there have been many trials on MAB. Since then, there have been many new marketing strategies from pharmaceutical companies. However, the question remains whether these agents actually make a difference?

## REVIEW OF LITERATURE

In 1989 the SWOG group looked at MAB and survival benefit and found a 24% benefit with leuprolide and flutamide versus leuprolide alone (NCI 0036). However they subsequently published a report the following year stating that orchiectomy and flutamide as maximal androgen blockade (MAB) therapy vs. orchiectomy alone, which significantly did not improve survival (NCI 0105).

In 1999, Bennet *et al*.,[5] studied nine published randomized control trials with 4128 patients. Their groups included six studies using Goserelein, one using leuprolide and four including orchiectomy patients. The antiandrogen used was flutamide in a dose of 250 mg three times a day. They found a statistically significant 10% prolongation of overall survival in patients with advanced prostatic cancer who received MAB with flutamide. The cost effectiveness of flutamide in 1999 was in the range of $ 47,500 to $ 60,900. This meta-analysis had its limitations. The analyses were based on individual study level results rather than patient level results. They were also unable to analyze information on LHRH agonists separately from the orchiectomy patients. The patients with minimal or severe metastasis disease did not have a subset analyses. Minimal disease was defined as the absence of metastases in the skull, ribs, long bones, and soft tissues excluding lymph nodes.

**Figure 1:**
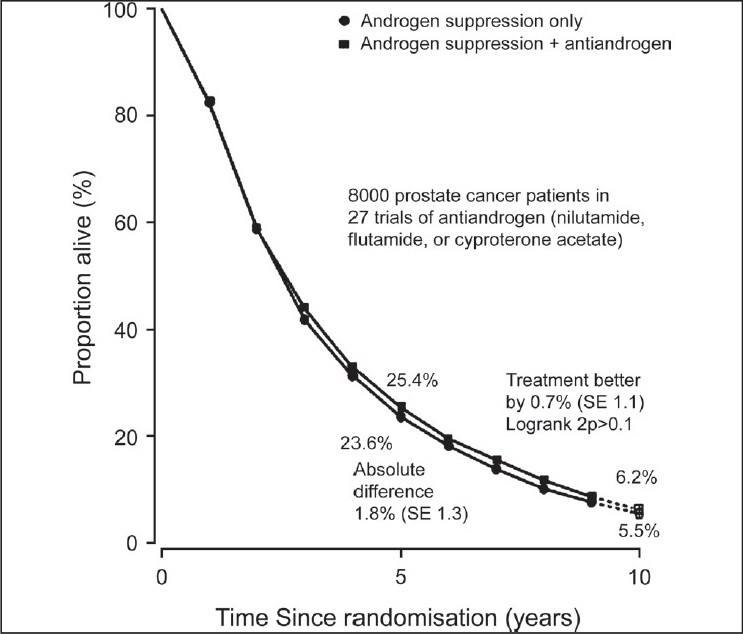
Ten-year survival in the 27 randomized trials of maximum androgen blockade versus androgen suppression alone, prostate cancer trialists’ collaborative group (Lancet 2000)

**Figure 2:**
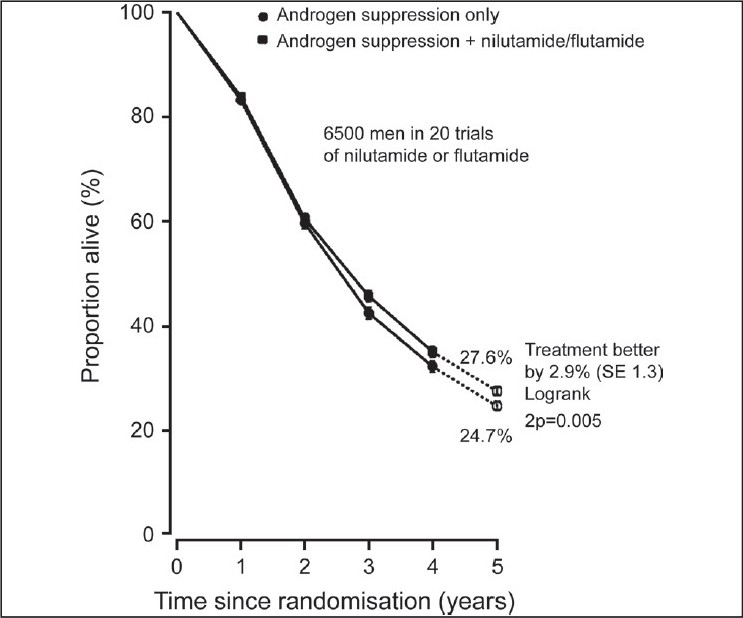
Five-year survival curves for 20 trials of androgen suppression plus nilutamide or flutamide versus androgen suppression alone, PCTG; Lancet 2000

**Figure 3:**
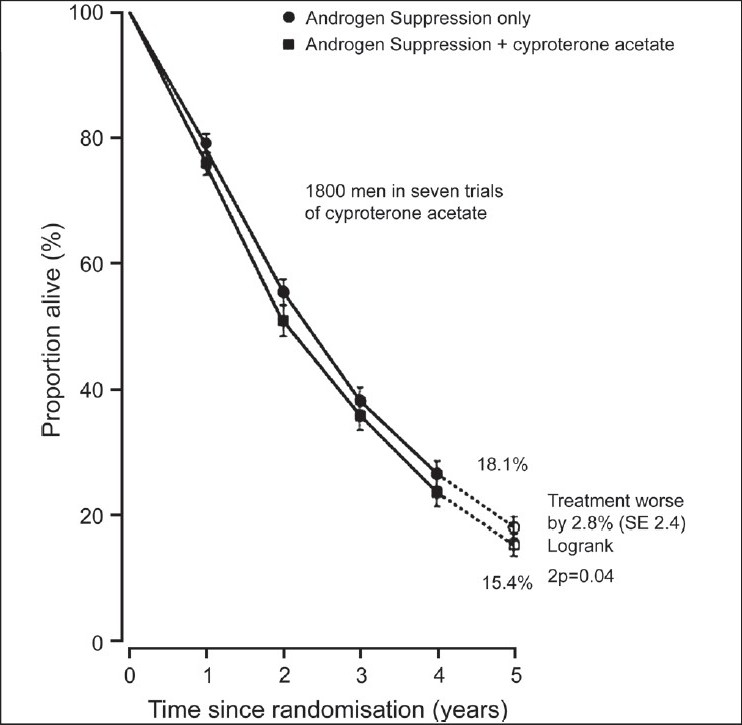
Five-year survival curves for seven trials of androgen suppression plus cyproterone acetate versus androgen suppression alone, PCTG; Lancet 2000

In 1995, the Prostate Cancer Trialists’ Collaborative Group (PCTCG)[6] published their first individual patient level meta-analysis comprising 5170 patients and 22 RCMAB trials. They updated their review published in Lancet[7] in 2000. Their review included individual patient data (IPD) from randomized controlled trials (RCTs) that began before December 1989. Studies that compared MAB with AS alone were eligible for inclusion. MAB was defined as AS plus immediate administration of an anti-androgen given for at least one year or until disease progression. Studies of men with advanced prostate cancer were eligible for inclusion. Men with metastatic (88%) and locally advanced (12%) cancer were included. The men's ages ranged from younger than 65 years to older than 75 years. Duration of survival was the main outcome of interest. The outcomes reported were overall mortality, five year survival, and an analysis of non-prostate cancer deaths. Data from 8,275 men in 27 RCTs were included. IPD could not be obtained for 183 participants in four other trials. The authors reported that the typical duration of follow-up was almost five years. Data for cause of death were obtained for only 20 of the 27 trials. There was no significant difference in overall mortality between metastatic and locally advanced disease, or between the age groups less than 65 years, 65 to 74 years, and 75 years and over, or according to whether AS was achieved by orchiectomy or drugs [Figure 1]. Based on 20 trials, 2,778 (80%) of the 3,475 deaths were attributed to prostate cancer. There was a non significant excess of non-prostate cancer deaths among men treated with MAB, but no association was found between this and age, stage, anti-androgen, or years of follow-up. Trials of nilutamide (eight RCTs, 1,688 men; n adjusted) or flutamide (12 RCTs, 4,803 men) showed an absolute increase in 5-year survival of about 3% with MAB [Figure 2], whereas trials of cyproterone acetate (37 RCTs, 1,784 men; n adjusted) showed a 3% decrease [Figure 3]. Some of the excess mortality among men treated with cyproterone acetate was accounted for by an excess of other deaths (i.e. not prostate cancer) in the cyproterone acetate trials, although non-prostate cancer deaths were not clearly significantly different between MAB and AS (2P = 0.05). This was the reason for cyproterone acetate falling out of favour as MAB.

In 1996 Boccon-Gibod[8] reviewed the literature on MAB and commented that there was marginal impact on overall survival. He also compared the various types of non-steroidal anti-androgens (flutamide, nilutamide and bicalatumide), and found no major difference apart from the spectrum of side effects.

The latest meta-analysis to review the effects of MAB was by Schmitt *et al*.,[3] for the Cochrane collaboration in the year 2003 and reviewed in 2008. They evaluated the relative efficacy of maximal androgen blockade on overall survival using any non-steroidal antiandrogens (NSAA) compared to castration alone (surgical or medical) for men with advanced prostate cancer. The secondary objectives were to evaluate the relative efficacy of maximal androgen blockade on progression-free survival and/or cancer-specific survival, overall survival, progression-free survival, and/or cancer-specific survival using any NSAA compared to castration alone (surgical or medical). They also reviewed the incidence of adverse effects from maximal androgen blockade.

**Figure 4:**
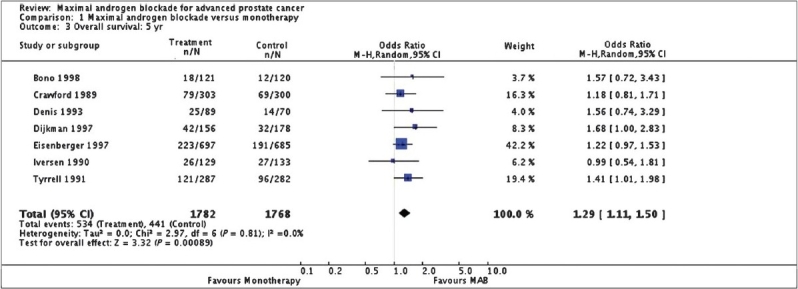
Maximum androgen blockade versus monotherapy; Overall survival at 5 years; Cochrane review

Three studies,[9–11] reported a statistically significant survival benefit that favored MAB with a five-year survival advantage ranging from 3% to 9%. The remaining trials reported no significant difference. The pooled estimate of the OR for overall survival progressively increased over: OR = 1.03 (95% CI: 0.85 to 1.25) at 1 year, OR = 1.16 (95% CI: 1.00 to 1.33) at 2 years, and OR = 1.29 (95% CI: 1.11 to 1.50) at 5 years [Figure 4]. The benefit from MAB was seen in patients with M 1 disease with absence of metastases in the skull, ribs, long bones, and soft tissues excluding lymph nodes.[9]

**Figure 5:**
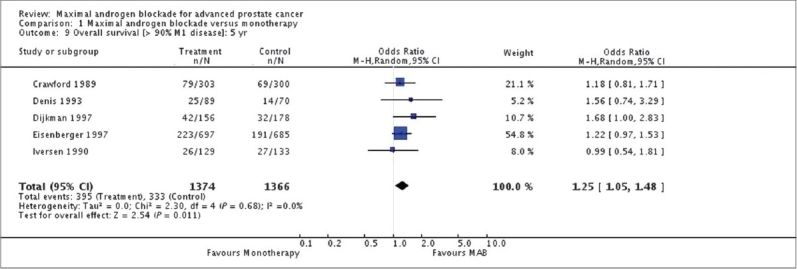
Maximum androgen blockade versus monotherapy; Overall survival at 5 years, > M1 disease; Cochrane review

When only studies with more than 90% M1 disease were included, the point estimate of the OR for overall survival was significant only at five years. The OR was 1.10 (95% CI: 0.86 to 1.41) at 1 year, 1.10 (95% CI: 0.92 to 1.32) at two years, and 1.25 (95% CI: 1.05 to1.48) at five years [Figure 5].

For the studies of MAB that utilized flutamide as the NSAA in comparison to orchiectomy the point estimate of the OR at two years favored orchiectomy over flutamide although the difference was not statistically significant. For those studies of MAB that incorporated nilutamide as the NSAA in comparison to orchiectomy, there was a statistically significant difference favoring MAB with nilutamide over orchiectomy.

**Figure 6:**
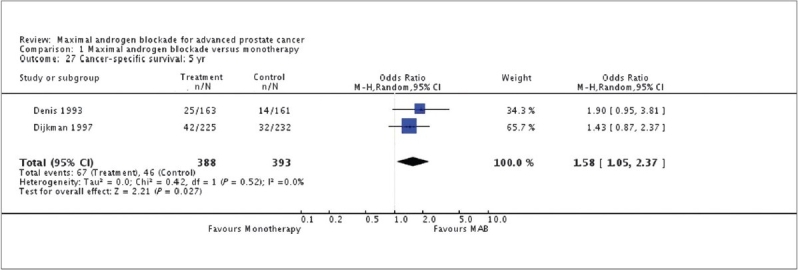
Maximum androgen blockade versus monotherapy; Cancer specific survival at 5 years; Cochrane review

The progression free survival pooled OR at one year was 1.38 (95% CI: 1.15 to 1.67), 1.19 (95% CI: 0.97 to 1.46) at two years and 1.14 (95% CI: 0.77 to 1.68) at five years. Cancer-specific survival progressively increased over time. The pooled OR of cancer-specific survival was 1.20 (95% CI: 0.92 to 1.57) at one year, 1.22 (95%CI: 0.86 to 1.73) at two years and 1.58 (95% CI: 1.05 to 2.37) at five years [Figure 6].

The major differences between those assigned to monotherapy with medical or surgical castration compared to those assigned to MAB included diarrhea (1.8%vs 9.7%), GI pain (1.6% vs. 7.4%), and non-specific ophthalmologic events (5.4% *vs* 29%). The occurrence of adverse events was more frequent with MAB than with monotherapy resulting in a withdrawal rate of 10% for those receiving MAB. Quality of life in the case of MAB and orchiectomy alone was addressed in only one study, with orchiectomy having a better quality of life.

The pooled estimates of the OR and RD increased in favor of MAB over time. However, the pooled OR and RD at one and two years were not statistically significant. Only the five-year follow-up observation significantly favored MAB. At five years, the pooled risk difference increased to an absolute difference of approximately 5%. The number of patients that needed treatment in order to save one life decreased from 31.3 at two years to 20.8 at five years. Adverse events were more frequent in those receiving MAB and resulted in withdrawal of therapy in more than 10% of the patients. Only 4% of those receiving monotherapy withdrew. Additionally, there was a reduction in the QOL in the first six months of MAB.

Bicalatumide was introduced in 1996 following a large randomized study[12] comparing bicalatumide with flutamide each in combination with LHRH analogs. At a median follow up of 49 weeks, the time to treatment failure was significantly longer for the bicalatumide patients compared with flutamide. After a median follow up to 95 weeks, bicalatumide, in combination with LHRH analog therapy produced at least equivalent efficacy in terms of survival.

### Maximal androgen blockade-The Asian data

There is very limited data from India and the rest of Asia on prostate cancer and maximal androgen blockade.

In 2001, Ansari *et al*.,[13] from the All India Institute of Medical Sciences published their data on eighty patients with advanced carcinoma prostate. This three year study compared orchiectomy alone to a combination of orchiectomy and flutamide. They concluded that the addition of flutamide to orchiectomy did not given any significant benefit to PSA changes as well the survival in advanced carcinoma of prostate.

In 2007, Hinotsu[14] *et al*., published their data on the trends in the treatment of carcinoma prostate in Japan. Patients who received MAB accounted for 59% of all patients. MAB being more often selected for patients who were rated as being at high risk on the basis of high Gleason score or PSA level upon diagnosis in each clinical stage of the disease.

In 2007, Usami *et al*.,[15] published their report on a Phase III double blind RCT comparing MAB with bicalatumide 80 mg in combination with LHRH as compared to LHRH alone. They showed favorable results for time to disease progression and time to treatment failure. The interim report on overall survival was not statistically significant.

### Ongoing trials

There are currently few trials regarding maximal androgen suppression; both being conducted in the United States. The first of these compares bicalatumide to bicalatumide with goserelin or orchiectomy.[16] This is a six year study started in 2001 and to be updated this year. The other trial is by the University of Washington on maximal androgen suppression in localized prostate cancer.[17] This study is currently recruiting participants.

### Side effects of antiandrogens

Nonsteroidal antiandrogens (bicalutamide, flutamide, nilutamide) competitively inhibit the binding of androgens to the androgen receptor; the serum testosterone levels are not suppressed and may even be raised.

Bicalatumide is the most extensively studied drug. The most frequently associated side effects are gynecomastia (70%) and breast pain. These may be prevented with the concomitant use of Tamoxifen or local radiotherapy. Hepatotoxicity, the most serious side effect of nilutamide, flutamide and cyproterone acetate is relatively uncommon with bicalutamide. Flutamide can produce fatal hepatotoxicity (1–5%) apart from diarrhea and gynecomastia. Both these drugs cause impotence in about 20 % of individuals. Nilutamide has a higher rate of impotence of 50%. The other associated effects not seen with the other antiandrogens but with nilutamide are visual disturbances (delayed adaptation to darkness, 33%) and alcohol intolerance (20%). Hepatotoxicity is rare (1%) with nilutamide.

Cypoterone acetate is associated with cardiovascular toxicity (4–40%), impotence (80%), lower testostrerone levels, loss of libido and hepatotoxicity (16%).

### The economic aspects of maximum androgen blockade

The cost of MAB is an issue which has its own implications. The lifetime cost of NSAA plus orchiectomy compared to orchiectomy alone was $20,700 and $7,000, respectively.[18]

In our country apart from the cost, there is the issue of availability. In India, nilutamide is not freely available. Cyproterone acetate (Androcur) is imported and costs about Rs. 68 for 50 mg.

Flutamide costs range from Rs. 27–30 per day. Bicalatumide is relatively expensive costing Rs. 27–42 for 50 mg.

## CONCLUSIONS

MAB has been around for more than 25 years. The issue as to its efficacy has been debated throughout. The steroidal anti-androgen cyproterone acetate has been shown to have an adverse effect when used for MAB.[6] Its role is probably limited to the prevention of the fl are reaction in patients on LHRH agonists. The non steroidal antiandrogens have a marginal benefit of increased overall survival by approximately 3% to 5% at five years. There may be a role for MAB in patients with metastatic carcinoma of prostate, low volume metastases, patients with M 1 disease with absence of metastases in the skull, ribs, long bones, and soft tissues excluding lymph nodes. The current European Association of Urology 2009 guidelines acknowledge the small survival advantage (less than 5%) with MAB as compared to monotherapy with the benefit being limited to patients taking non-steroidal anti-androgens for greater than five years.[19]

The spectrum of efficacy of the different non steroidal anti-androgens is almost the same, with the newer ones like bicalatumide, having a lesser degree of side effects. The disadvantage with them is the incidence of side effects and quality of life issues.

In a country like ours where follow up and rising costs of medical therapy are important issues, it may be worthwhile to consider orchiectomy as the first line of treatment for advanced carcinoma of prostate. The option of MAB can be utilized where cost effectiveness is not an issue.

## References

[1] Development of an Atlas of Cancer in India by ICMR, First All India Report 2001–2002. http://www.canceratlasindia.org/chapter3.

[2] Wadasadawala T, Murthy V, Mahantshetty U, Engineer R, Shrivastava S, Dinshaw K (2008). The European Organization for Research and Treatment of Cancer prostate-specific quality of life module (PR-25) in Hindi and Marathi: Translation and pilot testing process. J Can Res Ther.

[3] Schmitt B, Bennett C, Seidenfeld J, Samson D, Wilt T (2008). Maximal androgen blockade for advanced prostate cancer (Review). The Cochrane Library.

[4] Huggins C, Scott WW (1945). Bilateral adrenalectomy in prostate cancer: Clinical features and urinary excretion of 17-ketosteroids and estrogen. Ann Surg.

[5] Bennett CL, Tosteson TD, Schmitt B, Weinberg PD, Ernstoff MS, Ross SD (1999). Maximum androgen-blockade with medical or surgical castration in advanced prostate cancer: A meta-analysis of nine published randomized controlled trials and 4128 patients using flutamide. Prostate Cancer Prostatic Dis.

[6] (1995). Maximum androgen blockade in advanced prostate cancer: An overview of 22 randomised trials with 3283 deaths in 5710 patients. Prostate Cancer Trialists’ Collaborative Group. Lancet.

[7] (2000). Maximum androgen blockade in advanced prostate cancer: An overview of the randomised trials. Prostate Cancer Trialists’ Collaborative Group. Lancet.

[8] Boccon-Gibod (1996). Department of Urology, CHU Bichat, Paris, France. Maximum androgen blockade in 1996. Euro Urol.

[9] Crawford ED, Eisenberger MA, McLeod DG, Spaulding JT, Benson R, Dorr FA (1989). A controlled trial of leuprolide with and without flutamide in prostatic carcinoma. N Engl J Med.

[10] Dijkman GA, Janknegt RA, De Reijke TM, Debruyne FM (1997). Long-term efficacy and safety of nilutamide plus castration in advanced prostate cancer, and the significance of early prostate specific antigen normalization. International Anandron Study Group. J Urol.

[11] Denis LJ, Carnelro de Moura JL, Bono A, Sylvester R, Whelan P, Newling D (1993). Goserelin acetate and flutamide versus bilateral orchiectomy: A phase III EORTC trial (30853). EORTC GU Group and EORTC Data Center. Urology.

[12] Blackledge G, Kolvenbag G, Nash A (1996). Bicalutamide: A new antiandrogen for use in combination with castration for patients with advanced prostate cancer. Medical research Department, Zeneca Pharmaceuticals limited, Mereside, Macclesfield, UK. Anticancer Drugs.

[13] Ansari MS, Gupta NP, Hemal AK, Dogra PN, Seth A (2001). Orchiectomy versus combined androgen blockade in the management of advanced carcinoma prostate. Indian J Urol.

[14] Hinotsu S, Akaza H, Usami M, Ogawa O, Kagawa S, Kitamura T (2007). Current status of endocrine therapy for prostate cancer in Japan analysis of primary androgen deprivation therapy on the basis of data collected by JCaP. Jpn J Clin Oncol.

[15] Usami M, Akaza H, Arai Y, Hirano Y, Kagawa S, Kanetake H (2007). Bicalatumide 80 mg combined with a luteinizing hormone-releasing hormone agonist (LHRH-A) versus LHRH-A monotherapy in advanced prostate cancer: Findings from a phase III randomized, double-blind, multicenter trial in Japanese patients. Prostate Cancer Prostatic Dis.

[16] Randomized phase III step up study on initial androgen monotherapy in comparision watchful waiting in asymptomatic T 1–3 any Gleason, N 0 or N × M 0 prostate cancer treatment without local treatment with curative intent. NCT00014586.

[17] Maximal androgen suppression in localized prostate cancer (TAPS). NCT00298155.

[18] Seidenfeld J, Samson DJ, Aronson N, Albertson PC, Bayoumi AM, Bennett C (1999). Relative Effectiveness and Cost-Effectiveness of Methods of Androgen Suppression in the Treatment of Advanced Prostate Cancer. Evidence Report/Technology Assessment No. 4. (Prepared by Blue Cross/Blue Shield Association Evidence-based Practice Center under Contract No. 290–97-0015). AHCPR Publication No. 99-E0012. Rockville, MD: Agency for Health Care Policy and Research. Evid Rep Technol Assess (Summ).

[19] European Association of Urology (2009). Guidelines on prostate cancer.

